# Upper Airway Characteristics and Morphological Changes by Different MADs in OSA Adult Subjects Assessed by CBCT 3D Imaging

**DOI:** 10.3390/jcm12165315

**Published:** 2023-08-15

**Authors:** Nicolò Venza, Arianna Malara, Claudio Liguori, Paola Cozza, Giuseppina Laganà

**Affiliations:** 1 Department of Systems Medicine, University of Rome “Tor Vergata”, Viale Oxford 81, 00133 Rome, Italy; drariannamalara@gmail.com (A.M.); giuseppinalagana@libero.it (G.L.); 2 Sleep Medicine Centre, Department of Systems Medicine, University of Rome “Tor Vergata”, 00133 Rome, Italy; dott.claudioliguori@yahoo.it; 3 UniCamillus—Saint Camillus International University of Health Sciences, 00131 Rome, Italy; profpaolacozza@gmail.com

**Keywords:** OSAS, Mandibular Advancement Device (MAD), CBCT, upper airway

## Abstract

This study aimed to analyse the effectiveness of four different designs of the Mandibular Advancement Device (MAD) and the morphological changes on upper airway characteristics of Obstructive Sleep Apnea (OSA) patients inducted by each of them, detected by Cone Beam Computer Tomography (CBCT) 3D imaging. Twenty-two patients were recruited after an OSA diagnosis with PSG. Four different customised and titratable MADs were used and an initial CBCT scan was obtained for each patient. Six months after the end of the MAD titration phase, all the subjects performed a second PSG with the MAD in situ; the second PSG showed an Apnoea–Hypopnoea Index (AHI) of <5 or a decrease of 50% in AHI when compared with the initial AHI. Moreover, a second CBCT scan with the MAD in situ was performed. DICOM files were imported into the airway analysis software programme and the pharyngeal area around the oropharynx was highlighted. The area and volume of the oropharynx with and without the device was evaluated. A considerable improvement of the airway was observed (+33.76%), and a significant difference in the enlargement ratio between the posterior soft palate (+32.41%) and the posterior tongue (+36.96%) region was also found. The greatest increase in airway volume was achieved in patients treated with the MAD Forward and TAP (+42.77% and +41.63%, respectively). MAD therapy is effective to treat moderate to severe OSA with an increased upper airway volume. The design of the MAD can influence the effectiveness of the treatment.

## 1. Introduction

Obstructive Sleep Apnea (OSA) is a severe public health issue, resulting in high blood pressure, excessive daytime sleepiness and a compromised quality of life [1].

OSA is a common breathing disorder during the sleep characterized by snoring and recurrent collapse of the pharyngeal airway while sleeping, associated to a partial reduction (hypopnea) or complete cessation (apnea) of airflow lasting at least 10 s. Most breaks last between 10 and 30 s, but some may persist for a minute or more [2]. This can lead to relevant reductions in oxygen saturation in the blood, with oxygen levels that can decrease by up to 40% or more in more severe cases. The central nervous system responds to the lack of oxygen with an alert mechanism, causing a short awakening from sleep in order to restore normal breathing. This pattern can occur hundreds of times in a single night. The result is a fragmented sleep that often produces an excessive level of daytime sleepiness [3].

The interruption of breathing during sleep has many adverse health consequences, including cardiovascular diseases, collagenopathy, metabolic disorders like—insulin resistance and glucose metabolism—other chronic respiratory diseases, epilepsy, Alzheimer, neoplasms, kidney diseases and gastroesophageal reflux. However, the pathogenic mechanisms of OSAS in organs are complex and intertwined and not fully understood [4,5,6,7,8,9]. This disorder is the product of a complex interaction between anatomical factors (i.e., round airways, length and volume of the soft palate, length of the upper airways, pharyngeal fat deposits, adeno-tonsillar hypertrophy, tongue volume, class II skeletal profile and morphological deviations of the cervical spine), sleep-related factors and central nervous system control over ventilation [10].

The prevalence of OSA was assessed to be approximately 7 to 14% for adult men and 2 to 5% for adult women in a population-based study that used a cutoff of AHI ≥ 5 events/h (hypopneas associated with oxygen desaturations of 4%) associated with clinical symptoms to define OSA [11]. The most represented category of patients with OSAS are male individuals, obese and aged 65 or older. Unfortunately, OSA is still an under-diagnosed medical condition and more than 85% of patients with clinically significant OSA are never diagnosed [12].

Polysomnography is the gold standard test for the diagnosis of OSA in adult patients in whom there is a concern for OSA, based on a comprehensive sleep evaluation. It indicates a simultaneous recording of several physiological parameters during the night for the evaluation of the physiological and pathological phenomena that may occur while sleeping [11].

OSA management requires a long-term multidisciplinary approach. Therapeutic choices include lifestyle changes, drug therapies, mechanical or surgical therapies. Mandibular advancement devices (MAD) are a valid therapeutic alternative for a mild/moderate OSA and for patients with severe OSA who do not comply or do not respond to Continuous Positive Airway Pressure (CPAP). In particular, MADs interfere with the mandible and tongue, with the pharyngeal dilator muscles and, indirectly, with the soft palate. By displacing the mandible forward, these structures that constitute the lumen of the oropharynx are also stretched forward, increasing upper airway space and reducing the collapsibility of the pharynx [13,14]. According to the possibility of modifying the occlusal relationship, the MADs are divided into two categories: titratable and not titratable. The titrators have a mechanism that allows an increase to the degree of advancement of the jaw during the course of therapy. Therapy with advancing jaws has a beneficial effect on cardiovascular comorbidities, endothelial function and cognitive and psychomotor performance [15,16,17]. Although the use of the MAD is widely described in the literature, no guidelines regarding the choice of the type of device and the titration protocol are available to date. Furthermore, its effects on the upper respiratory tract, especially when examined in 3D, are still not fully described.

Over the years, different imaging modalities were proposed to directly describe the status of the upper airway, the risk of OSA and the effectiveness of MAD to increase the airway space. Moreover, several studies in the literature have tried to define the role of cephalometric skeletal models in identifying the predisposition to generate the syndrome.

Cone Beam Computer Tomography (CBCT), with its low effective radiation dose and low scanning time (10–70 s), represents an effective technique for a 3D complete evaluation using a large field of view protocol for a comprehensive head and neck evaluation [18,19,20].

According to the most recent research on OSA and 3D airway analysis, the oropharynx area is one of the most common measurements to assess the presence and severity of OSA. A high chance of severe OSA was noticed in patients with an upper airway area less than 52 mm^2^, an intermediate chance with an upper airway area between 52 mm^2^ and 110 mm^2^, and a low chance with an upper airway area larger than 110 mm^2^ [20]. Therefore, CBCT can be an important tool to evaluate the efficacy of MAD in terms of mechanical modification of oropharyngeal tissues. More information on how the different types of mandibular advancement devices modify the upper airways could guide the choice and their clinical management. Furthermore, as far as we knew, there were no studies comparing more than two different type of MAD and their effects on airway changes.

The primary objective of this study was to evaluate the effectiveness of four different designs of MADs in subjects with moderare to severe OSAS. The second aim was to analyse the morphological changes in the oropharynx airway inducted on the subjects by the different types of mandibular advancement devices using CBCT 3D imaging.

Our hypotheses were that: (1) the MADs are effective for the treatment of moderate to severe OSAS; (2) there are significant differences in the modifications induced on oropharyngeal tissue by different MAD designs.

## 2. Materials and Methods

This study followed the principles laid down by the World Medical Assembly in the Declaration of Helsinki 2008 on medical protocols and ethics, and it was approved by the Ethical Committee of University of Rome “Tor Vergata” (protocol number 0015966/2019). Written consent was obtained from all subjects included in the study.

The study group was composed of 22 OSA patients, aged between 30 and 70 years old. Specialists in sleep medicine referred all patients after a nocturnal polysomnography test and a diagnosis of OSA. The diagnosis of OSA was based on the recognized criteria, including an apnoea–hypopnoea index (AHI) of ≥5/h per hour during sleep [2].

We considered the following as inclusion criteria: age > 18 years; first-line treatment for moderate OSA with mild to moderate daytime sleepiness and without severe cardiovascular comorbidities or a second-line treatment for moderate to severe OSA after intolerance or refusal of positive airway pressure therapy; signed informed consent.

The following were excluded from the study: patients with a central apnoea index ≥5/h; severe sleep comorbidities other than OSA; any contraindication for MAD evaluated by a dental specialist investigator (periodontal disease, less than 10 teeth in both dental arches), history of temporomandibular disorders such as pain and limited opening to less than 40 mm; a coexisting psychiatric or neuromuscular condition that could affect compliance; occupational hazards such as professional drivers; and pregnant or lactating women.

For each subject, the following information was recorded: age, gender, and BMI. Moreover, every participant responded to the Epworth Sleepiness Scale.

Four customized and titrabled MAD, registered with the Gorge Gauge with an initial mandibular advancement of 60% maximal jaw protrusion without muscular pain for patients was used and the titration was adjusted at subsequent visits.

For each patient, after establishing the diagnosis of OSAS through PSG and the therapeutic indication for the MAD, a CBCT was performed and the impressions were taken for the manufacture of the MAD. After the delivery of the MAD, the good adaptation of the patient to the device was established; the titration process started. Once the titration process was accomplished, follow-up visits were planned. The same process was employed for each MAD device in order to align the level of mandibular advancement when the titration phase was completed. At the follow-up visit, a clinical examination was performed to assess OSA symptoms, compliance and side effects.

During the titration phase, 3 of the 22 participants recruited had slight symptoms of temporo-mandibular junction (TMJ) dysfunctions. For these patients, titration was slowed down to enable the progressive adaptation to the device. At the end of titration and subsequent monitoring, no patients showed any signs or symptoms of TMJ. The titration phase ended when the patient showed a significant reduction in symptomatology (supported by ESS score) in the absence of TMJ signs or symptoms. A second PSG with the MAD in situ was performed at the end of the titration step.

The therapeutic effect of MAD was judged positive when it showed an AHI of <5 or a decrease of 50% in AHI in the presence of MAD when compared with the AHI changes in the absence of MAD.

The MAD used for each subject was randomly selected. All subjects of the sample reached excellent therapeutic results.

Six months after the end of the titration phase, a second CBCT scan with a MAD in situ was performed.

The MADs selected for the study were chosen as representatives of the different types of MAD most commonly used by the clinicians and best described in the literature. MADs actually differ from one another for the different materials for construction, protruding mechanism, anchoring mechanism, titratable mood and vertical dimension.

The characteristics of the MADs used are summarized in Table 1.

The Silensor SL device is composed of two thermoformed splints covering the upper and lower arch, respectively. These two plates are connected by a fixed joint in the upper part at the level of the canine, and in the lower part at the level of the lower first and second molars. Direction of the connector enables the movement of the mandibular protrusion to be defined.

The inter-incisal gap between the two plates is between 2–4 mm. This device is provided by six connectors with 1 mm increment lengths. This device additionally enables limited lateral and opening movements of the mouth. In this study, 5 subjects used the Silensor SL MAD.

The TAP device consists of two acrylic plates that cover the upper and lower arches, respectively. These two splints are joined by an anterior traction: a fixed mechanical hinge and an inseparable pivot. The fixed hinge is connected with an advancer screw which allows 7 mm of maximum elongation. The screw activation allows very small and precise increments. Each complete activation produces a 0.25 mm advancement. The interincisal distance between the 2 plates is between 4–6 mm. This device also allows wide laterality movement, but no mouth opening movements. Six subjects were randomly selected for this MAD.

The Telescopic Advancer Deviceconsists of two fitted acrylic plates joined by lateral connectors composed of a tube and piston mechanism (called Herbst attachments) with the possibility of adjusting the protrusion very accurately; each ¼ activation produces a 0.1 mm advancement with 7 mm of maximum elongation. The interincisal distance between the two plates is between 6–8 mm. This device also allows wide laterality and opening movements.

Five subjects were selected in our sample to use the Telescopic Advancer Device.

The Forward device consists of two fitted acrylic plates connected by buccal flanges with a metal core angled at 70°. The screw mechanism with an in-built stop is present in the upper plate to allow for 7 mm of maximum elongation with 0.1 mm increments. The vertical opening between the two plates is between 6–8 mm. This device also allows for a limited opening and no mouth laterality movements. From the initial sample, 6 subjects were selected to use the Forward MAD device.

**Figure 1:**
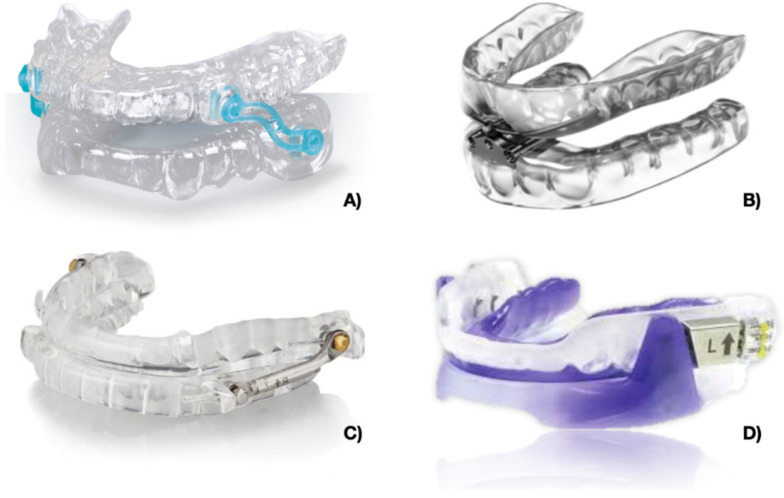
MADs used in the study. (**A**) Silensor SL; (**B**) TAP; (**C**) Telescopic Advancer; (**D**) Forward.

The MADs used in the study are shown in Figure 1.

**Figure 2:**
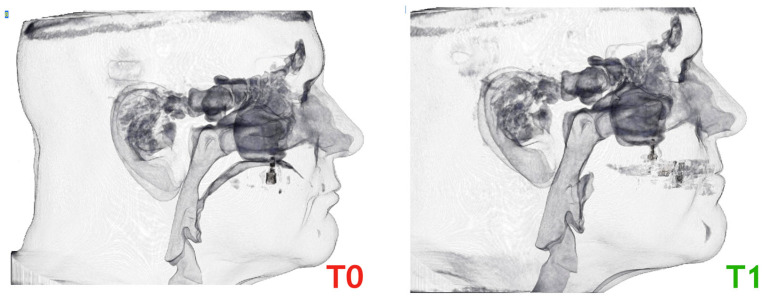
CBCT scans before the treatment (T0) and at the end of the titration with the MAD in situ (T1).

All CBCT examinations were performed with the NewTom VGi EVO unit (NewTom 3G, QR s.r.l.; AFP Imaging, Elmsford, NY, USA). Each patient was placed upright and seated, and the head was fixed such that the Frankfurt plane was parallel to the floor, teeth were into occlusion, avoiding tongue movements, without swallowing or respiratory movements during inspiration at rest. All CBCT acquisitions were made by the same operator. Two scans were obtained—the first one (T0) before the therapy, and the second after 6 months to the end of the titration phase with the MAD in situ (T1) (Figure 2).

The CBCT images were exported as DICOM files (.dcm) and then imported into the airway analysis software programme. A coding system was used to anonymise the data.

To check the accuracy of the measurements, 7 patients were randomly selected whose images were re-analyzed after an interval of 14 days by the same operator; the intraclass correlation coefficient (ICC) was used for statistical analysis of the difference between the duplicate measurements. The agreement between the measurements was substantial (Kappa > 90).

After the PAS segmentation process, the three-dimensional models were cut out. The pharyngeal area around the oropharynx, where the origin of the apneas is mainly located, was considered.

The plane between the base point (Ba) and the posterior nasal spine point (PNS) represented the cranial margin of the PAS. The caudal margin was described as a plane parallel across the tip of the epiglottis (E). All PAS models were divided into an upper and a lower part by a parallel plane passing through the lowest point of the soft palate (P).

Noise in the airway slices was minimized while the airway volume was selected.

**Figure 3:**
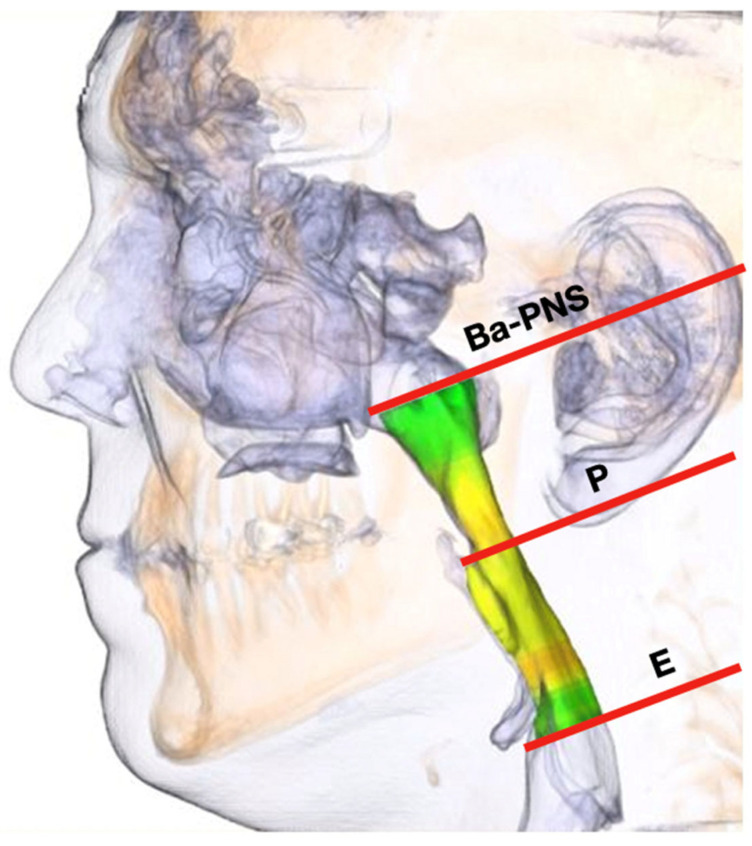
Schema as defined for each measurement region on the upper airway form 3-dimensional CBCT reconstruction.

To measure the area and volume of the oropharynx with and without the device, it had to be divided into 2 parts: the posterior soft palate airway (Ba–PNS–P), and the posterior tongue oropharynx (P–E) (Figure 3).

The cross-section evaluation was carried out on the upper and lower shear surface of the three-dimensional PAS models. Once the corresponding cut surface was marked, the software calculated the exact cross-sectional area.

**Figure 4:**
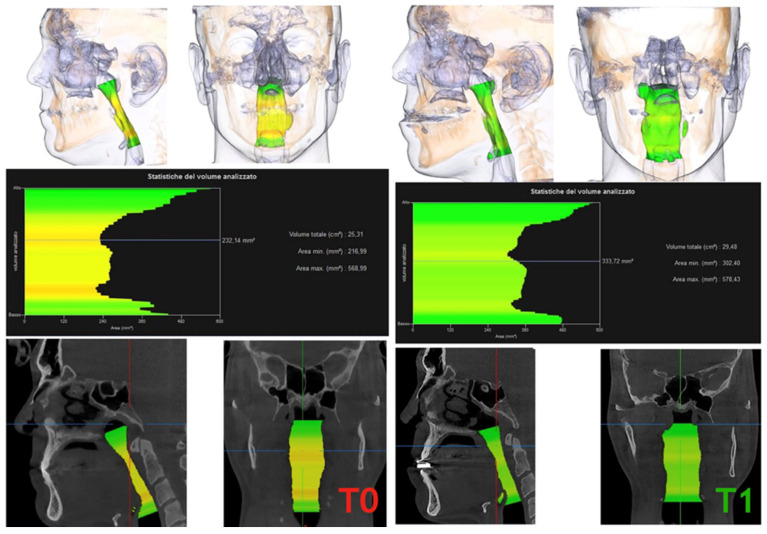
3D comparison of area and volume at T0 and T1.

In addition, the volume of the imported three-dimensional PAS models (upper and lower PAS) was calculated by the software (Figure 4).

All the measurements of the airway were performed by a study co-investigator who was blinded to the subject’s apnea condition and was evaluated for reliability with repeated measurements for n = 10 after 1 week under the same conditions. Inter-rater reliability was also evaluated for n = 10 by a second co-investigator of the study.

### Statistical Analysis

A summary of all parameters was made in terms of averages, standard deviations and ranges. Comparison of the means of each parameter before and after treatment was conducted using paired *t*-tests assuming the normality of the data. In addition, the relationship between AHI and different parameters was assessed using Pearson’s correlation analysis. The statistical significance was evaluated at the 5% level.

## 3. Results

The sample was composed of 22 OSA patients (13 M, 9 F). The mean age was 54 years (±7.7) with an average body mass index (BMI) of 27 (±4.3) kg/m^2^. At the baseline, the average AHI/h was 31.7/h (±9.8). The MAD was well tolerated by all patients. No significant differences were observed in the severity, frequency or duration of the reported side effects between devices.

At the end of the titration period, there were no significant differences in mandibular protrusion between the different four MAD devices (mean elongation 6.3 ± 1.3 mm). The mean AHI/h with the MAD in situ was 11.97 (±12.8). Concerning the different types of devices, it could be noted that, considering the polysomnography indexes, the results overlap almost with all the devices used (Table 2). Compared to the baseline, the oropharyngeal airway volume increased substantially with all devices—on average by 36.96% ± 19.22%, *p* = 0.003. In relation to the different type of device, it was possible to note that with the use of the MAD Forward, an average variation oropharyngeal airway volume of 45.48% was obtained; with the TAP, an achievement of 43.89%; with the Silensor, the mean increase was 33.02%; finally, with the Telescopic Advancer, the effectiveness was lower with an average increase of 25.46% Table 3.

## 4. Discussion

This study aimed to analyze the effectiveness of four different designs of MAD and the morphological changes on upper airway characteristics of OSA patients inducted by each of them, detected by CBCT 3D imaging.

Our study, in line with the most recent review, showed significant results in terms of respiratory indexes and the oropharyngeal airway volume with all types of MAD [21].

The optimum vertical dimension and mandibular protrusion of an oral appliance required to achieve a successful treatment outcome in patients with OSA was an issue of debate in literature. Gupta et al. [22], in their work, analyzed four different non treatable MADs (60% of protrusion and 4 mm of vertical opening, 60% of protrusion and 6 mm of vertical opening, 70% of protrusion and 4 mm of vertical opening, and 70% of protrusion and 6 mm of vertical opening), evaluating respiratory indexes and assumed that the best configuration was 70% of protrusion and 4 mm of vertical opening. In our study, only titratable MADs were considered, and even if the initial mandibular protrusion was at least 60% of the maximum protrusion, the final position of the MAD was reached by increasing the protrusion to the maximum value tolerated by the patient in the absence of muscle pain. This made it possible to obtain a clear improvement in respiratory indexes for all patients.

Secondly, concerning how the vertical dimension can influence the effectiveness of MAD, Pitsis et al. [23] made a comparison of the effects of two MAD setups (4 mm vs. 12 mm opening, both with similar mandibular protrusion). No significant differences in treatment efficacy were reported by the authors, but the patients’ compliance was higher for the device with the smaller interincisal opening. By contrast, Ye Min Soe et al. [24] suggested that the increased vertical distance between the maxilla and mandible, due to the appliance, affected the exacerbation of the respiratory status during sleep.

In line with Pitsis et al.’s results [23], the vertical dimension of the devices we considered, ranging from 2 to 8 mm, did not significantly affect the polysomnographic results and oropharyngeal volumes achieved at the end of the titration in our study.

Furthermore, regarding the protruding mechanism which mainly differentiates the MADs used in this study, our results showed better polysomnographic and volumetric results for the MAD Forward and TAP compared to the Silensor and Telescopic advancer. Literature concerning the protruding mechanism appeared still restricted and, as far as we knew, there were no contributions comparing different MADs’ protruding mechanisms.

The optimal criteria for the construction of a MAD that can achieve the best therapeutic results are the subject of wide discussion in the literature. Numerous studies propose a mandibular protrusion that varies between 60 and 80% of the maximum protrusive of the patient, but there is no agreement on a value that can be the optimal. In our study, the initial protrusive was 60% each, but the final position was achieved by progressive progression through the MAD titration. We can affirm that, as stated in the literature, the best degree of progress is the maximum achievable in the absence of pain or discomfort muscle or joint for the patient [25,26].

Several studies have highlighted the superiority of bi-block MADs over mono-blocks [27] and customized MADs over prefabricated MADs [26], but, to our knowledge, as we stated before, there were no studies in the literature comparing the different types of the MAD protrusion mechanism. The best results have been highlighted with the use of MAD, whose protrusion mechanism allows adequate and constant support of the jaw and soft tissues even during the natural mandibular movements that can occur during the night. In particular, the Forward device, with its long buccal fins, allows the maintenance of the protruded position of the jaw even during the small movements of the mouth, avoiding a posterior mandibular rotation that would reduce the volume of the airways. The TAP, with its front hooks, prevents any opening movement of the jaw. Finally, the Telescopic advancer and the Silensor do not have any mechanism that prevents the opening and post-rotation of the jaw. However, the effectiveness of the MAD cannot be standardized for all patients and although some technical characteristics of the device make them more effective, there is no perfect MAD for everyone. The characteristics of the patients must be carefully evaluated and studied in order to use the best device that can be not only effective in the short term, but which can guarantee long-term compliance. The “patient factor” ultimately includes both anatomical and physiological as well as psychological aspects.

It would be interesting to reassess long-term CBCT outcomes with 5- and 10-year follow-ups. This would make it possible to understand if, with the same initial satisfactory clinical results, a greater increase in oropharyngeal volumes can reduce the incidence of recurrence of the disease by supporting soft tissues more effectively. As described in the literature from the existing few long-term studies, the long-term use of a MAD by patients depends on its tolerability and the dental effects that determine over the years.

In literature, only a few studies compared the effects of different MADs on the upper airway volume using three-dimensional imaging. Suga et al. compared two MADs (one semi-rigid and one rigid devices) and reported no significant differences in upper airway volume with either device examined [28]. Most studies that compared two or more MADs have evaluated polysomnographic [29] and cephalographic [30] parameters as primary findings. Lateral cephalograms offer a two-dimensional assessment of upper airway anatomy. The main limitation of cephalograms is that they only allow anteroposterior comparisons of the upper airway.

By using this imaging procedure, Brown et al. found that MADs primarily increased pharyngeal lateral dimensions [31]. CBCT delivers three-dimensional images and a more accurate overview of the upper airway [32].

Because no scientific research study is perfect, this study has its limitations. The CBCT images were not taken in supine position but in the upright position and the patients were awake. The position of the head and the phase of respiration cannot be controlled completely. As the upper airway is a dynamic organism, variations in respiratory artefacts, swallowing, and the position of the head may affect the measurements. Despite thorough and exact indications before the cone-beam CT scan, these biases must be taken into account. Another limitation is related to the sex of patients. A larger proportion of male to female patients can skew results. The final limitation was the small number of participants. A long-term prospective study with a large number of patients would be desirable to investigate MAD-inducted changes in the posterior airway space in more detail.

## 5. Conclusions

All the customized and titratable MADs analyzed led to a significant improvement of the polysomnography’s indexes in patients with mild to severe OSAS. We can thus affirm that a customized and titrable MAD is a valid therapeutic option for the treatment of moderate to severe OSAS. Careful patient selection and titration protocol with progressive increments may help to achieve optimal clinical outcomes while maintaining patient compliance and avoiding potential side effects.

The type of MAD Forward, consisting of acrylic plates supported by angled side flanges, and the type of MAD TAP, consisting of acrylic plaques joined by an anterior traction, resulted in a greater increase in the volumetric size of the oropharynx.

The vertical dimension of the MAD did not significantly influence the effectiveness of the treatment with an adequate mandibular protrusion. However, it was recommended not to exceed the 8 mm of interincisal opening to facilitate better acceptance of the device by the patient.

The results of this study showed that CBCT is an important test for the diagnosis and treatment choice of OSA patients. The authors recommend the evaluation of CBCT images, including the calculation of respiratory tract measurements, as part of the overall assessment.

Future CBCT studies on the incidence of recurrence and soft tissue adaptation after prolonged use of MAD are desirable to assess whether, with the same initial satisfactory clinical results, mandibular advancement devices that leads to a greater volumetric increase of the airways are more effective in counteracting the phenomena of recurrence of pathology.

## Figures and Tables

**Table 1:** Main characteristics of the devices used.

	Silensor SL	TAP	Telescopic Advancer	Forward
**Material**	Biocompatible thermoforming material	Biocompatible thermoforming material	Fitted acrylic trays	Fitted acrylic trays
**Protruding mechanism**	Lateral traction: six replaceable connectors with different lengths	Anterior traction: a fixed mechanical hinge and inseparable pivot	Lateral compression: plug and tube components (Herbst attachments)	Lateral compression: buccal flanges angled 70° in the lower tray and screw in the upper one
**Titrable mood**	Changing adjustable connectors	Advancer screw in the upper plug	Advancer screw in the plug	Advancer screw in the upper plug
**Amount of protrusion**	5 mm of protrusive range with 1 mm increments	7 mm of maximum elongation with 0.25 mm increments	7 mm of maximum elongation with 0.1 mm increments	7 mm of maximum elongation with 0.1 mm increments
**Vertical opening**	2–4 mm	4–6 mm	6–8 mm	6–8 mm

**Table 2:** Respiratory indices detected at T0 and T1.

			T0			
MAD	AHI/h	RDI	ODI	Av. Ox. Sat	Lower Ox. Sat	Sat. <90%
**All**	31.71	31.50	29.11	89.54%	76.13%	19.63%
**Forward**	32.87	32.70	28.44	87.41%	71.29%	18.37%
**Telescopic Advancer**	28.48	28.61	24.87	93.32%	84.46%	21.11%
**Silensor**	30.76	30.68	29.29	90.24%	74.65%	20.01%
**TAP**	31.38	31.49	30.09	89.81%	76.75%	19.24%
			**T1**			
**MAD**	**A** **HI/h**	**RDI**	**ODI**	**Av. Ox. Sat**	**Lower Ox. Sat**	**S** **at. <90%**
**All**	11.97	14.10	8.93	93.98%	90.33%	7.56%
**Forward**	13.78	13.87	11.12	91.34%	89.43%	9.45%
**Telescopic Advancer**	11.34	11.09	9.54	94.05%	89.99%	8.32%
**Silensor**	14.43	12.45	9.04	93.58%	91.42%	7.87%
**TAP**	9.82	10.12	7.98	95.34%	90.31%	6.85%

**Table 3:** Volumetric increase of the oropharynx from T0 to T1.

	Ba–PNS–E		Ba–PNS–P		P–E	
MAD	Average	SD	Average	SD	Average	SD
**All**	33.76%	33.73%	32.41%	25.56%	36.96%	19.22%
**Forward**	42.77%	26.90%	40.04%	19.11%	45.48%	32.41%
**Telescopic Advancer**	21.53%	4.87%	19.27%	1.24%	25.46%	8.11%
**Silensor**	29.11%	7.33%	31.76%	18.96%	33.02%	21.18%
**TAP**	41.63%	6.88%	38.58%	23.12%	43.89%	9.18%

## Data Availability

The data presented in this study are available on request from the corresponding author.
